# MiRNA in cervical cancer: Diagnosis to therapy: Systematic review

**DOI:** 10.1016/j.heliyon.2024.e24398

**Published:** 2024-01-12

**Authors:** Hiwot Tezera Endale, Yitbarek Fantahun Mariye, Habtu Kifle Negash, Fethiya Seid Hassen, Wastina Bitewlign Asrat, Tiget Ayelgn Mengstie, Winta Tesfaye

**Affiliations:** a Department of Biochemistry, School of Medicine, College of Medicine & Health Sciences, University of Gondar, Ethiopia; b Department of Human Physiology, School of Medicine, College of Medicine & Health Sciences, University of Gondar, Ethiopia; c Department of Human Anatomy, School of Medicine, College of Medicine & Health Sciences, University of Gondar, Ethiopia; d Department of Obstetrics & Gynecology, School of Medicine, College of Medicine & Health Sciences, Addis Ababa University, Ethiopia

**Keywords:** miRNAs, Cervical cancer, Biomarker, Metastasis, Therapeutics

## Abstract

Cancers are one of the most public health problems worldwide. Among them, cervical cancer (CC) is the fourth most prevalent cancer with 604 000 new cases and 342 000 deaths. Mostly, it is associated with Human papillomavirus (HPV). It has been caused by the aggregation of genetic and epigenetic modifications in cervical epithelial cells. Although genetic mutations are given great attention for the carcinogenesis of CC, epigenetic changes have emerged as a hotspot area for CC biomarkers research with great implications for early diagnosis, prognosis, and treatment response prediction of the disease. Recently, there are several studies focused on miRNAs as biomarkers of cervical cancer. However, the precise function of miRNAs in the development of cervical cancer is not still completely understood, particularly when it comes to unconventional sampling materials like cervical mucus and plasma serum. Hence, this review article will give a summary of the miRNAs profiles that emerge at different stages of cervical cancer progression and their downstream effects on target genes and associated signaling pathways. Finally, these results may provide insight into the use of miRNAs as biomarkers for the prediction or diagnosis of cervical cancer or the development of miRNA-based therapeutics against cervical cancer.

## 1 Background

Cancers are one of the most public health problems worldwide. Among them, cervical cancer is the fourth most prevalent cancer in women worldwide [1]. 604 000 new cases and 342 000 deaths of cervical cancer were reported globally in 2020 [2]. Human papillomavirus (HPV) induces invasive cervical cancer by transforming the normal cervical epithelium into a preneoplastic cervical [3]. Chronic infections may progress into cervical intraepithelial neoplasia (CIN) grade 1 to 3 intraepithelial lesions. Cervical cancer is a preventable and curable disease if it is diagnosed early. Although it is curable, it remains a cause of death in women from third-world countries as they seek help after they develop signs, symptoms, and advanced complications [4,5].

Recently, some studies showed the association between epigenetics and cervical cancer development and progression. Epigenetics is heritable changes in gene expression without a permanent change in the DNA sequence and plays a crucial role in the pathogenesis of tumors. Disruption of epigenetic processes can lead to genomic instability and mutation. Consequently, the genetic mutations allow epigenetic regulator modification and initiate the development and progression of carcinogenesis [[6], [7], [8], [9]]. Epigenetic changes include DNA methylation, hydroxymethylation, demethylation, chromatin remodeling, histone modification, and regulation by RNA-mediated targeting regulators can affect the expression of genes without modifying genomic DNA sequence [10,11].

MicroRNA (miRNA) is a small non-coding RNA molecule of 20–30 nucleotides that binds to target messenger ribonucleic acid (mRNA) that does not encode a protein but inhibits post-transcriptional gene expression. MicroRNA is important in the regulation of gene expression, metabolism, apoptosis, neuronal development, and cell cycle. There are two types of miRNAs, oncogenic miRNAs and tumor suppressor miRNAs which are involved in carcinogenesis. Many studies have revealed its role in cancer diagnostic markers and as anticancer therapy. Cervical cancer tissue examination revealed that abnormal expression of a large number of miRNAs plays crucial roles in tumorigenesis, progression, and metastasis of the disease [[12], [13], [14], [15], [16]]. Cancer involves genetic and epigenetic alterations that can up or down-regulate miRNA levels. Studies showed that miRNAs alter the expression levels of cervical cancer cells associated with HPV. Altered miRNA expression profiles have also been reported in cervical cancer [[17], [18], [19]]. However, the precise function of miRNAs in the development of cervical cancer is not still completely understood, particularly when it comes to unconventional sampling materials like cervical mucus and plasma serum. The purpose of this review is to summarize the miRNA profiles that emerge at different stages of cervical cancer progression and their downstream effects on target genes and associated signaling pathways. Finally, these results may provide insight into the use of miRNAs as biomarkers for the prediction or diagnosis of cervical cancer or the development of miRNA-based therapeutics against cervical cancer.

### 1.1 Biogenesis of miRNA and how it controls target genes

RNA polymerase II/III transcripts are processed post and co-transcriptionally in the nucleus to start the synthesis of miRNA [20]. The majority of miRNAs that are currently known are intragenic, produced mostly from introns and a few of them from exons; however, some are intergenic and are independently transcribed under the control of their promoters [21,22]. Sometimes a family of miRNAs is formed from the processing of a single long transcript with comparable seed regions. There are canonical and non-canonical pathways for miRNA biogenesis. The primary miRNAs are transcribed from their genes and processed into precursor miRNA (pre-miRNA) by a microprocessor complex made up of an RNA binding protein called DiGeorge Syndrome Critical Region 8 (DGCR8) and a ribonuclease III enzyme called Drosha. This process is known as canonical miRNA biogenesis [23].

The RNase III endonuclease Dicer processes the pre-miRNA to create mature miRNA, which is composed of 21–23 nucleotides, after being delivered to the cytoplasm by an Exportin 5 (XPO5)/RanGTP complex [24,25]. In the end, a miRNA-induced silencing complex is formed when 5p or 3p strands of mature miRNA duplex are ATP-dependently loaded into the Argonaute (AGO) family of proteins (AGO1-4 in humans). Either Drosha/DGCR8-independent pathways or Dicer-independent pathways are used to process non-canonical miRNAs. Small hairpin RNA (shRNA), which is processed by the microprocessor complex to create pre-miRNA, is transported to the cytoplasm with the aid of an XPO5/RanGTP complex in the Dicer-independent miRNAs pathway. Additionally, the pre-miRNA is covert into mature miRNA inside the cytoplasm in a way that is dependent on AGO2 but independent of Dicer. The spliceosome and the XPO1 complex, transport mirtrons and 7- methylguanosine (m7G) capped pre-miRNA respectively, to the cytoplasm, both of which are Drosha/DGCR8-independent [26,27]. The mature miRNA is then processed by Dicer-dependent cleavage within the cytoplasm. In the end, each process results in the production of a miRISC complex that is functional. To silence genes, miRISC attaches to mRNA and causes either translational repression or mRNA degradation/deadenylation. These pathways most likely cooperate and are controlled differently depending on the gene or cell type [28].

## 2 Human papillomaviruses

**Fig. 1:**
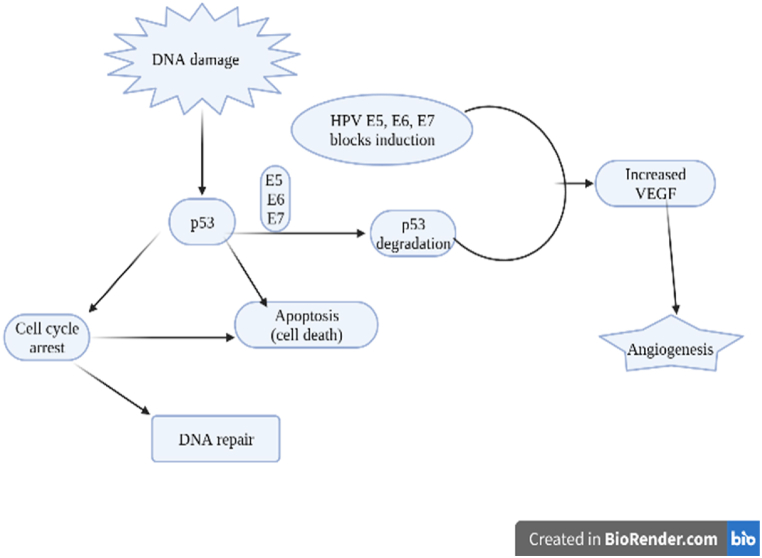
E5/E6/E7 signaling pathways of the human papillomavirus (HPV).

HPVs are Papillomaviridae family members with an 8 kb non-enveloped double-stranded circular DNA (dscDNA) genome. In comparison, HPVs are resistant to heat treatment up to 56 °C, chemical solvents, and desiccation. Since HPV can only develop in humans, molecular methods are commonly employed to identify it as the only known host. Ten open reading frames (ORFs) are located in three genomic locations on the one strand of the dsDNA genome, which serves as a transcriptional template. Virus replication and transcription require the proteins E1, E2, E4, E5, E6, and E7, which are encoded in the early (E) region. Virion assembly requires the structural capsid proteins L1 and L2, which are encoded in the late (L) region. The long control region (LCR) or upstream regulatory region (URR) is a non-coding section that makes up the third region of the HPV genome. It contains *cis*-elements that contain a transcriptional regulatory sequence, a DNA replication origin, and one or more promoters that govern the expression of E6 and E7 oncoproteins. Increased transcription of the oncogenes E6 and E7 is a characteristic of invasive cervical carcinomas and is essential for the development of the cancer caused by HPV. A number of tiny proteins (around 150 amino acids) that are encoded by the E6 ORF have the potential to self-regulate the E6 promoter (p97), which is in charge of regulating the expression of these proteins. The cell cycle is disrupted as a result of physical interactions between E6 and a variety of cellular components produced from the host (Fig. 1). The HPV E6 protein frequently attaches to and inhibits the activity of p53, triggering ubiquitin-dependent degradation of p53 with E6 associated protein (E6AP), decreasing p53's half-life from 3 h to 20 min and losing its biological capabilities. The usual control of p53 by mdm-2 is replaced in cells infected with HR-HPV by the development of the E6-p53-E6AP-complex. In contrast, E7, which is in charge of cell proliferation, binds to pRB protein to release E2F transcription factor and control the transition of the cell cycle from the G1 to the S phase, which is followed by DNA replication and ultimately leads to cell division. Additionally, E7 ″LR-HPV” is less effective at binding to pRB than E7 ″HR-HPVs,” and it is ineffective in assays for cell transformation and ras oncogene [[29], [30], [31]].

### 2.1 miRNA's part in the development, progression, and dissemination of CCs associated with HPV

**Fig. 2:**
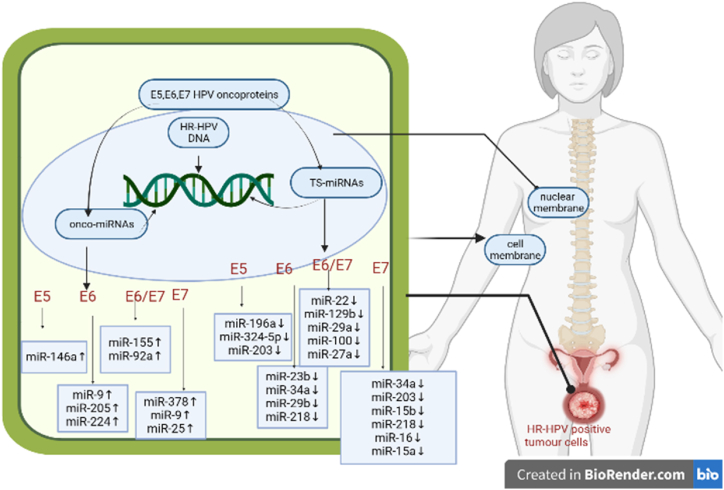
Modulation of miRNAs in cervical cancer by HR-HPV E5, E6, and E7.

The HPV oncoproteins (E5, E6, and E7) can suppress the expression tumor suppressive miRNAs in the host cells, according to a comparison of miRNA expression in HPV positive and negative CC (Fig. 2). While certain miRNAs control carcinogenesis at every stage, others are only vital during the transition from CIN2/3 to invasive CC (ICC) as shown in Table 1 [[32], [33], [34], [35], [36], [37], [38], [39], [40], [41]] (see Table 2).

**Table 1:** Role of miRNAs in tumor development, progression, and metastasis of CC.

List of miRNA with tumor suppression role		
miRNA	Target	Their effect on biological function
7	FAK	↓migration, ↓invasion [32]
10b	IGF1R	↓proliferation, ↓migration, ↓invasion [32]
23b	six1, ALDH1A1, c-Met, Zeb1	↓EMT, ↓migration, ↓invasion [32]
29a	CDC42/PAK1	↓proliferation, ↓migration [32,33],
99a/b	mTOR	↓ Cell growth, ↓invasion [33]
100	PLK1	↓cell growth, ↓proliferation, ↑ apoptosis [34]
101	Cox-2, JAK2	↓proliferation, ↓migration, ↓invasion, ↑apoptosis [32]
124–3p	IGF2BP1	↓proliferation, ↓migration, ↓invasion [35]
125a	STAT3	↓ EMT, ↓proliferation, ↓migration, ↓invasion [32,36]
125b	STAT3, p53, BAK	↓ EMT, ↓proliferation, ↓migration, ↓invasion [33,41]
126	ZEB1, MMP2, MMP9, Bcl2l2	↓proliferation, ↓migration, ↓invasion [32]
133a	NEAT1, EGFR, SOX4	↓proliferation, ↓migration, ↓invasion, ↑apoptosis [33]
138	c-Met, hTERT	↓proliferation, ↓migration, ↓invasion [34]
143	Bcl-2, KRAS, RREB1, GOLM1	↓proliferation, ↓migration, ↓invasion, ↑apoptosis [33,37]
144	VEGFA, VEGFC	↓ migration, ↓invasion [35,36],
145	KRAS, RREB1	↓motility, ↓invasion [35]
148a	RRS1, NFkB1	↓proliferation, ↓migration, ↓invasion, ↑apoptosis [35]
183	ITGB1, MMP9	↓proliferation, ↓migration, ↓invasion [35]
195	CCND2, MYB, Smad3, HDGF	↓proliferation, ↓migration, ↓invasion [36]
203	VEGFA, IRF1, BANF1	↓ tumor growth ↓proliferation, ↓migration, ↓invasion
205	IGF1R	↓proliferation, ↓invasion [36]
214	GALNT7, Bcl2l2, FOXM1	↓proliferation, ↓migration, ↓invasion, ↑apoptosis
215–3p	SOX9	↓proliferation [36]
218	SFMFBT1, DCUN1D1, IDO1, LYN, NF-kB	↓Cell viability, ↓ EMT, ↓migration, ↓invasion, ↑apoptosis [36]
302	DCUN1D1	↓migration, ↓invasion [36]
320	FOXM1, Mcl-1	↓Viability, ↓migration, ↓invasion [36]
342–3p	FOXM1	↓proliferation, ↓migration, ↓invasion [33,37]
372	CDK2, cyclin A1	↑Cell cycle arrest [32,37]
424	Chk1, *p*-Chk1	↑Cell cycle arrest [38]
491–5p	hTERT	↓proliferation, ↓migration, ↓invasion, ↑apoptosis [39]
494	SOCS6	↓proliferation, ↓invasion [40]
497	IGF1R	↓proliferation, ↓invasion [40,41]
544	YWHAZ	↓proliferation, ↓migration, ↓invasion [41]
**list of miRNAs that participate in tumor development, progression, and metastasis**		
**miRNA**	**Target**	**Their effect on biological function**
10a	CHL1	↑cell growth, ↑migration, ↑invasion [56]
17–5p	TGFBR2, CCND1	↑Proliferation, ↑metastasis [57]
18a	STK4, YAP	↑cell proliferation [58]
19a/b	CUL5	↑cell growth, ↑invasion [59]
20a/b	TNKS2, ATG7, TIMP2	↓proliferation, ↓migration, ↓invasion [60]
21	PTEN, RASA1, TIMP3, AP-1	↑proliferation, ↑invasion, ↓apoptosis [61]
27a/b	B4GALT3, DGCR8, PLK2	↑EMT, ↑proliferation, #apoptosis [62]
31	ARID1A, BAP1	↑EMT, ↑proliferation, ↑migration, ↑invasion [63]
92a	FBXW7, p21, DKK3	↑cell viability, ↑proliferation, ↑invasion [64]
93–5p	THBS2, MMP-2, MMP-9	↑proliferation, ↓migration, ↓invasion [65]
106a	LKB1, TIMP2	↑Proliferation, ↑migration, ↑invasion, ↓autophagy [66]
129–5p	Shh, ZIC2, CXCL1, Gli1, Gli2, Ang2, VEGF	↓proliferation, ↓migration, ↓invasion [67]
130a	TIMP2	↑cell proliferation, ↑invasion [68]
146a	HPGD, IRAK1, TRAF6	↑Cell viability, ↑cell proliferation, ↑migration, ↑invasion [69]
155	LKB1, TP53INP1	↑Proliferation, ↑growth, ↑migration,↑invasion, ↓apoptosis [70]
181a	PRKCD, INPP5A, OPN	↑proliferation, ↑invasion, ↓apoptosis [70]
181b	AC9	↑proliferation, ↓apoptosis [71]
196a	FOXO1, p27(Kip1)	↑proliferation [71]
221	PTEN, TWIST2, THBS2, MeCP2, MBD2	↑proliferation, ↑migration, ↑invasion [72]
224	RASSF8	↑proliferation, ↑migration, ↑invasion [73]
590–5p	CHL1	↑cell growth, ↑migration, ↑invasion [74]
629–3p	PTP4A1, ERK1/2	↑cell proliferation, ↑migration, ↑invasion [75]
1246	THBS2	↑cell proliferation, ↑migration, ↑invasion [76]

A list of miRNA, miRNA target genes, and their effect on cellular functions and CC disease processes, are listed. ↑: induces/increases; ↓: inhibits/reduces; CC: cervical Cancer.

**Table 2:** Expression status of miRNA during cervical cancer progression.

miRNAs	Expression status	Chromosome	Location
miR-375	Downregulated	2	2q35 [56]
miR-203	Downregulated	14	14q32.33 [56]
miR-34a	Downregulated	1	1p36.22 [57]
miR-21	Upregulated	17	17q23.1 [57]
miR-25	Upregulated	7	7q22.1 [58]
miR-106a	Upregulated	X	Xq26.2 [59]
miR-10a	Upregulated	17	17q21.32 [60]
miR-23b	Downregulated	9	9q22.32 [61]
miR-100	Downregulated	11	11q24.1 [62]
miR-145	Downregulated	5	5q32 [63]
miR-185	Upregulated	22	22q11.21 [64]
miR-20b	Upregulated	X	Xq26.2 [65]
miR-31	Upregulated	9	9p21.3 [66]
miR-155	Upregulated	21	21q11.3 [67]
miR-424	Downregulated	X	Xq26.3 [68]
miR-193b	Downregulated	16	16p13.12 [69]
miR-497	Downregulated	17	17p13.1 [70]
miR-185	Upregulated	22	22q11.21 [71]
miR-92b	Upregulated	1	1q22 [72]
miR-93	Upregulated	7	7q22.1 [73]
miR-146a	Upregulated	5	5q33.3 [74]
miR-200a	Upregulated	1	1p36.33 [75]
miR-27a	Upregulated	19	19p13.12 [76]

### 2.2 miRNA dysregulation in cervical cancer

Numerous systems regulate the expression of miRNA, and disruptions thereto can lead to the onset of various diseases, including cancer. Alterations on the miRNA biogenesis proteins, promoters, sequence, target sites, or biogenesis regulatory regions can all result in dysregulation, loss of specificity, or even suppression of the miRNA function, which can have detrimental effects on the cell [35].

A wide range of studies have delineated a portion of the mechanisms underlying miRNA dysregulation and their pattern of expression in cervical cancer. Strong evidence points to the possibility that genetic differences, such as genetic deletions, amplifications, or mutations, at the miRNA genomic loci could cause changes in miRNA expression. For instance, a large number of dysregulated miRNAs were found in advanced CC cells linked to increased Drosha expression and chromosome 5p gain. In clinical samples, these miRNAs include miR-31, miR-141, miR-203, which are elevated, and miR-193a-3p, which are downregulated. Furthermore, only five miRNAs were found to be down-regulated in Drosha-overexpressing cells, while the bulk of miRNAs were up-regulated [36]. It is widely recognized that aberrant DNA methylation modification of miRNA genomic loci and changes to transcriptional activators and repressors frequently result in an aberrant miRNA expression profile in cancer. The dysregulation of the proteins implicated in miRNA biogenesis, in addition to genomic alterations and patterns of methylation of the miRNA gene sequence, contributes to the alterations of miRNA expression in cervical cancer [37,38]. Single nucleotide polymorphisms (SNPs) are another factor that alters the expression of miRNA in cervical cancers. SNPs inside miRNA genes disrupt the secondary structure of the miRNA precursor, affecting strand choice, Drosha and Dicer processing, and ultimately changing the maturation process of miRNA genes. As a result, SNPs in miRNAs alter how well they attach to their target genes by deregulate their expression. These changes are strongly linked to the emergence of cancer [[39], [40], [41]].

### 2.3 Epigenetic and miRNA regulation in cervical cancer

Epigenetic phenomena are defined as changes in genomic DNA and chromatin that affect gene expression and are somatically heritable without involving changes in DNA sequence. DNA methylation is a post-replicative DNA modification that takes place on the 5′ position of cytosine rings found in CpG dinucleotides [42]. Cervical cancer cannot be caused solely by HPV; precancerous lesions and cervical cancer exhibit DNA methylation, which may play a role in the progression of cervical cancer. Many epigenetic changes, such as DNA hypo-methylation, hypermethylation of tumor suppressor genes, and histone modifications, are seen in cervical cancer [43]. DNA methyl transferase (DNMTs) is an enzyme that methylates DNA. The DNMTs-catalyzed reaction is reversible and preserves the methylation prototype throughout every cell division. DNMTs, which are mostly found in CpG islands, attach a methyl group to carbon 5 of cytosine residues that are next to guanine residues (5′ CpG 3′). MicroRNAs (miRNAs), which are non-coding genes, can also be regulated by methylation [44]. Transfection of cells with HPV 16 methylated genomes in vitro demonstrated transcriptional repression of DNA. Cervical cancer is associated with a number of methylation-regulated transcription factors, including p53, UTF1, paired box 1 (PAX1), and TWIST1 [[45], [46], [47], [48]].

The two most common DNA modifications seen during the early stages of cancer development are hypermethylation in the CpG island regions of tumor suppressor gene promoters and global hypomethylation in repetitive regions of DNA. DNA hypermethylation has been linked to the silencing of tumor suppressor genes in cervical cancer. Carcinogenesis has also been linked to the silencing of tumor suppressor miRNAs via hypermethylation of CpG islands in their promoter regions. Research findings indicate a significant correlation between elevated expression of DNMT1 mRNA or protein and low levels of serum folate and cervical carcinogenesis. The host's genome integrating high-risk HPV DNA, such as HPV 16 and 18, is a crucial step in the development of cervical cancer. Once the HPV 16, 18 DNA has incorporated into the human genome, it becomes methylated [[48], [49], [50], [51]].

High-risk HPV (HR HPV) contributes to the dysregulation of miRNA gene methylation in cervical cancer. According to certain theories, HR HPV may alter the methylation pattern of miRNA promoters [52,53]. Histones bound to promoter regions regulate gene expression through post-translational modification, which in turn controls the accessibility of transcription factors and the basal transcription machinery. Among the various modifications included are acetylation, methylation, phosphorylation, ubiquitination, and sumoylation. Numerous histone-modifying enzymes, including sirtuins, histone acetyltransferases, and histone deacetylases (HDACs), regulate the expression of tumor suppressor genes, oncogenes, and growth factors (HATs). Consequently, epigenetic regulation of gene expression results from dysregulation and mutation of histone modifier expression [54,55].

### 2.4 Aberrant expression of miRNAs in cervical cancer

Tumors frequently exhibit aberrant miRNA expression. MiRNAs is linked to cancer genesis, progression, metastasis, and prognosis in addition to controlling apoptosis, cell cycle progression, proliferation, and differentiation [[56], [57], [58]]. MiR-21, one of the up-regulated miRNAs, has been linked to the progression of CIN and ICC [34,59,60]. One of the most frequently expressed miRNAs in mammals, miR-21, has a demonstrated association with various cancers [61]. A crucial signal managing the balance between pro-inflammatory and immune-suppressive conditions, the miR-21 has been proven to be a vital regulator in cell survival and proliferation and is also associated with inflammation [61,62]. MiR-21 expression may have increased due to both E6 and E7. Ras-MeK-ERK pathway, which is controlled by PTEN tumor suppressor, a target of miR-21, can feed back on viral E6 and E7 [63,64]. This was supported by the finding that the miR-21 promoter region had more STAT3 and p65 NF–B binding in cervical cancer tissues [[65], [66], [67]]. In addition, miR-9, miR-25, miR-16, miR-106a, miR-10a, miR-185, miR-20b, miR-31, miR-92a, miR-155, miR-185, and miR-196a, miR-92b, miR-93, miR-146a, miR-378, miR-200a, and miR-27a are among the overexpressed miRNAs in the cervical carcinogen. The expression level of miR-27a increased in CIN stages and ICC, and it is an oncogenic miRNA that is controlled by p53, E2F, and c-Myc [58]. ZEB1, ZEB2, TGFB2, and EXOC5 are miR-200a targets that have been linked to the metastatic potential [68]. Squamous cell carcinomas (SCC) had considerably lower levels of miR-196a than CIN2/3 despite showing an increased expression from CIN1 to CIN2/3 [33]. Some miRNA clusters, including the miR-221/222 and miR-17-92 clusters, may be tumor-suppressive (TS) in one type of cancer while being oncogenic in another. The following examples demonstrate the idea that miRNAs can function as either tumor suppressors or oncogenes, depending on the target genes and tissue context. MiR-221 and miR-222 may be regarded as tumor suppressors because they prevent erythropoiesis by preventing the oncogene c-KIT from being expressed. On the other hand, the upregulation of miR-221 and miR-222 is a component of the miRNA progression signature in liver tumorigenesis. In a mouse model of liver cancer, both miRs promote the growth of tumors and the colony formation of liver cancer cells in vitro. In a sophisticated study by Pineau, early immortalized progenitors were transduced with a liver-specific miR-221-expressing vector, after which mouse p53 liver progenitors were immortalized by c-MYC expression. Mice injected with miR-221-transduced hepatic progenitors showed reduced tumor free survival rates when compared to empty vector controls. A tumor suppressor and crucial regulator of mTOR kinase signaling at both the mRNA and protein levels, DDIT4's direct downregulation via miR-221 contributed to the suppressive effect. Furthermore, recent studies show that miR-221/222 directly suppresses at least six additional tumor suppressors, including p57, PTEN, TIMP3, p27, BIM, and FOXO3, in various cellular contexts by encouraging the proliferative and invasive phenotypes typical of blatantly malignant cells. These studies indicate that the PTEN-PI3K-AKT-mTOR axis, which is related to the development of tumors in the liver, lung, and breast, is another level of disruption caused by the miR-221/222 cluster. They also demonstrate that a single miRNA may have a potent inhibitory effect on the entire cellular pathway due to its capacity to modify multiple genes involved in the same pathway [67,68].

In tissues infected by high-risk HPV and CIN2 or CIN3, miR-218 was found to be the most significantly downregulated miRNA [68,69]. In CIN and ICC, miR-29a expression was also downregulated [70]. The CDC42-PAK1 signaling pathway was demonstrated to be the target of miR-29a, which was found to limit cell proliferation and migration and protect against cervical cancer [71]. Additionally, the methylation of SOCS1, a tumor suppressor, by the miR-29a could prevent cervical cancer from spreading [72]. Additionally, miR-375, miR-99a, miR-203, miR-125b, miR-34a, miR-23b, miR-100, miR-145, miR-424, miR-193b, and miR-497 were found to be downregulated in cervical cancer. Some miRNAs showed dysregulation throughout the entire carcinogenesis process, while others only showed changed expression levels between CIN2/3 and ICC [73,74]. As of right now, E6 and E7 are most likely responsible for HPV's regulation of miRNA expression (Fig. 2), and E6's degradation of p53 helps to reduce miR-28b and miR-34a expression [75]. By specifically targeting cyclin E1, the miR-497 was able to decrease cervical cancer HeLa cells [76]. These miRNAs have the potential to become serum panels that serve as novel, non-invasive biomarkers for the detection of cervical cancer.

In conclusion, we have gathered and analyzed the evidence regarding the involvement of miRNA in cervical cancer and how miRNA deregulation affects the malignant transformation of cervical cancer, as well as its targets that can be used for both prognostic and therapeutic techniques. The current review includes thorough information about differentially expressed miRNA from recently published studies, which have been linked to the advancement of cervical cancer. In terms of targets, more than 40 miRNAs have undergone experimental validation. They have also been studied for their mechanisms and roles in various cervical cancer stages.

### 2.5 miRNAs regulating signaling pathways in CC

Since miRNAs affect their target genes, they are essential for the initiation, spread, and metastasis of cancer. Understanding the relationship between target genes and miRNAs is crucial to understanding the regulatory mechanisms of miRNAs in tumor growth, metastasis, and invasion. The miRNAs have an impact on the initiation and progression of tumors and are essential for the regulation of post-transcriptional genes. Research has demonstrated that the metastasis of CC is facilitated by a wide variety of signaling pathways. The development and metastasis of CC cancer have been connected to numerous signaling pathways. The phosphatidylinositol 3-kinase/protein kinase-B (PI3K/AKT) signaling pathway is one such pathway that is crucial for the migration and invasion of cancer cells and is recognized as a major driver in carcinogenesis. Hedgehog, p38/MAPK, wnt/β-catenin, and p53 are some other examples of these pathways. Moreover, dysregulated miRNAs can be differentiated from oncogenic miRNAs and TS miRNAs. Several biological processes have been linked to both classes of miRNAs, including the invasion, growth, and progression of CC. According to new research, miRNAs regulate target genes and are involved in a wide range of processes, including the initiation and progression of cancer. As a result, they may function as oncogenes or antioncogenes. The following pathway linked the different miRNA expression patterns in CC to complex CC progression and oncogenic or TS effects [77,78].

EGFR signaling pathways controls a number of signaling channels. Through EGFR downregulation, miR-875–5p decreases CC cell tumorigenesis and metastasis. On the other hand, overexpression of miR-155 inhibits the epithelial-mesenchymal transition (EMT) brought on by EGFR signaling, which in turn reduces CC cell invasion and migration [79,80].

The JAK/STAT pathway is crucial for signal transduction and cell division. MiR-9 functions as TSmiRNA in CC by targeting Interleukin-6 (IL-6) and thereby suppressing the Jak/STAT3 pathway. Furthermore, miR-126 functions as a TS in CC cells, blocking the expression of matrix metalloproteinases (MMP2, MMP9) and zinc finger E-box binding homeobox 1 (ZEB1), which in turn suppresses the JAK2/STAT3 signaling pathway. It's possible that miR-211, which prevents CC proliferation, also targets ZEB1. Moreover, miR-146 targets STAT3 to limit cell invasion and proliferation in CC [[81], [82], [83]].

Tissue homeostasis and cell proliferation depend on notch signaling. By focusing on the KDM5B gene, upregulation of miR424–5p inhibits Notch1 and Notch2 expression, which in turn inhibits cell proliferation. However, by inhibiting urokinase plasminogen activator (UPA) and thereby deactivating the Notch1 and Jagged1 signaling pathways, miR-34a reduces the invasiveness of cervical cells. Conversely, the downregulation of miR-628–5p activates Jagged 1, which in turn promotes the growth of cancer cells [[84], [85], [86]].

Wnt/β-catenin signaling pathway-miR-135a directly inhibits the proteasomal degradation of β-catenin, it upregulates Wnt/β-catenin, which has an oncogenic role in CC. Furthermore, Dickkopf-1, an inhibitor of the Wnt/β-catenin signaling pathway, is inhibited by the upregulation of miR-372 and miR-373, which promotes the invasion and multiplication of CC cell lines. MiR-146a suppresses invasion, migration, and proliferation by specifically targeting the Wnt/β-catenin signaling pathway's intermediate gene, CTNNB1. MiR-4524b-5p overexpression is seen in CC tissues and it targets WTX, also known as APC membrane recruitment protein 1, which is involved in the degradation of β-catenin, to increase invasion and migration. The promotion of vascular endothelial growth factor (VEGF) expression through upregulation of miR-9–5p leads to an increase in angiogenesis and proliferation. This is achieved by reducing the activity of E-cadherin, which in turn activates the β-catenin signaling pathway by targeting cytokine signaling. Whereas [[87], [88], [89], [90], [91]].

An essential adhesion kinase involved in cell invasion and survival is FAK. Through its targeting of FAK, the protein kinase B (AKT) upstream, miR-7 prevents CC invasion and metastasis. Furthermore, miR-133b inhibits MST2, cell division control protein 42 homolog (CDC42), and ras homolog gene family member A (RHOA) while stimulating AKT and extracellular signal-regulated kinase (ERK1 and ERK2), members of MAPK signaling pathways. Conversely, upregulating MAPK1 expression through downregulation of miR-497–5p promotes the advancement of CC. Furthermore, miR-205 has been shown by microarray analysis to stimulate angiogenesis and cervical cell invasion by binding to TS lung cancer 1 (TSLC1) and activating the AKT signaling pathway. Through the inhibition of tripartite motif-containing 3 (TRIM3), the miR-454–3p increases cell growth and inhibits apoptosis by activating p38 MAPK signaling and down-regulating the expression of cleaved caspase-3 and p53. On the other hand, CC cell migration and proliferation are inhibited by overexpressing phosphatase and tensin homolog (PTEN). By suppressing PTEN expression, miR-21 is upregulated in invasive CCs, facilitating invasion, migration, and proliferation. MiR-23b-3p overexpression prevents cervical cell migration and proliferation by deactivating FAK, the downstream target, and c-Met. Furthermore, by suppressing the expression of the MAPK signaling pathway-activated ETS domain-containing protein Elk-1 (ELK1), miR-326 prevents the growth of cancer cells [[92], [93], [94], [95], [96], [97], [98], [99]].

### 2.6 Diagnosis of cervical cancer using miRNA

Since RNA quickly degrades when transported into the blood by RNases, it was once believed that the miRNA cannot be identified in serum. Later, it was observed that secreted miRNAs were embedded in exosomes and granular vesicles that circulated in the circulation [100]. Again, increased expression of the miRNAs miR-21, miR-27a, miR-34, miR-34a, miR-146a, miR-155, miR-196a, miR-203, and miR-221 was observed, with miR-21, miR-27a, miR-34, miR-34a, and miR-196a being particularly highly expressed in SCC of the cervix. This suggests that miRNA levels in serum can be used for cervical cancer diagnosis. Besides miRNA-126, miRNA-20, miRNA-451 and miRNA-144 expression were high in cervical cancer. Aberrant hypermethylation is the cause of the alteration in miR-124. Additionally, methylation-specific PCR (MSP) and coupled bisulfite restriction analysis (COBRA) can be used to examine aberrant hypermethylation of miRNAs. Therefore, patients suspected of having cervical cancer can be diagnosed using the aberrant hypermethylation of miR-124 shown by MSP [101]. Additionally, high-grade dysplasia caused by HPV positivity has been linked to hypermethylation of miR-149, miR-203, and miR-375, and miR-203 and miR-375 may be indicators of precancerous abnormalities in the uterine cervix [102]. When used in combination with miRNA, it may be more appropriate to diagnose cervical cancer at various stages.

### 2.7 Role of miRNAs in therapeutic response to CC

Variable miRNA expression is up or downregulated in cervical cancer and can affect how responsive the disease is to radiotherapy and chemotherapy. It has been demonstrated that miR-138, miR-210, and miR-744 expression levels can improve sensitivity to ACA and cisplatin [103]. Additionally, the miR-214 can increase sensitivity to cisplatin by inhibiting Bcl2l2 expression, which can accelerate apoptosis and decrease cell growth by upregulating the proteins Bax, caspase-9, caspase-8, and caspase-3 [104]. Through the AKT-mTOR signaling pathway, miR-218 can improve sensitivity to cisplatin in cervical cancer [105]. MiRNA may, in the meanwhile, make cervical cancer treatments less effective. It was discovered that miR-375 helps cervical cancer cells develop paclitaxel resistance [106]. Another illustration is miR-181a, which has been demonstrated to boost cellular resistance to irradiation by suppressing radiation-induced apoptosis and reducing G2/M stage block and proapoptotic protein kinase (PRKCD) expression. When paclitaxel-resistant miRNA is overexpressed or when miRNA expression is particularly susceptible to cisplatin, cisplatin may be the best option. Such a therapeutic approach may be beneficial for cervical cancer molecularly targeted therapy or individualized treatment.

### 2.8 Immunotherapy approaches for the treatment of cervical cancer

A promising strategy is immunotherapy, a type of biological therapy that focuses on using living things, their byproducts, or the body's immune system to fight tumors in an immunosuppressive tumor microenvironment. To increase tumor reduction, reduce the risk of relapse, and extend a patient's life, various immunotherapy treatment modalities are being utilized. These can be used alone or in conjunction with other traditional cancer therapies like chemotherapy and radiation therapy. Scientists have reported a recent advancement in cancer research immunotherapy, based on a clinical trial in which every patient experienced a full remission of their colon cancer. Twelve people, who were all carriers of the same cancer mutation known as mismatch repair-deficient colorectal cancer, participated in this small-scale study. According to a study, 5–10 % of patients with colorectal cancer have this mutation. These patients' cancer tumors had not responded well to traditional chemotherapy or radiation treatment. Dostarlimab is a monoclonal antibody that increases T cells' sensitivity to identifying and eliminating cancer cells by binding to programmed cell death protein (PD-1) on their surface. In reaction, cancer cells create these molecules that bind to and inhibit PD-1 in order to conceal themselves from immune system penetration. Dostarlimab worked by assisting the immune system in identifying cancer cells, thereby reducing the likelihood that these cells would elude the body's defenses. Immunotherapeutic Methods for Cervical Cancer Treatment includes cancer vaccines, oncolytic virus therapy, adoptive cell therapies, and immune checkpoint inhibitors [107,108].

### 2.9 Role of TME-associated miRNAs in regulating drug resistance

In clinical practice, resistance to targeted therapy and chemotherapy presents a significant challenge. A growing body of research indicates that TME-derived miRNAs could impact drug resistance. Exosomal miR-122 (122-Exo) derived from MSCs derived from adipose tissue can be introduced into HCC cells to increase their chemosensitivity through cyclin G1 expression inhibition, metalloproteinease 10 disintegrin and inhibition, and insulin-like growth factor receptor 1 (IGF1R) expression inhibition. The antitumor efficaciousness of sorafenib in vivo against HCC was considerably enhanced by intratumoral injection of 122-Exo. Furthermore, sorafenib, a chemotherapeutic agent, can suppress the expression of miR-101 and increase the expression of dual-specificity phosphatase 1, which will prevent the growth of HCC that is induced by macrophages. Through the control of the PTEN/phosphoinositide 3-kinase/AKT signaling pathway, miR-21 was able to significantly reduce the sensitivity of GC cells to cisplatin chemotherapy both in vitro and in vivo. In GC, miR-21 was transferred from macrophages to GC cells via exosomes. Patients with esophageal cancer had serum levels of miR-27a/b that were significantly higher than those of healthy volunteers. It was also demonstrated that high expression levels of miR-27a/b induced the transformation of NFs into CAFs by upregulating TGF-β, which in turn caused esophageal cancer cells to become chemoresistance. These findings imply that miRNAs generated from TMEs are essential for drug resistance [109,110].

### 2.10 Future perspectives and conclusion

Considering whether miRNA signatures are connected to current and future cervical risk in the context of cervical cancer screening may require combining them with additional biomarkers. The analysis of miRNA levels in exfoliated cervical cells undoubtedly creates new opportunities for research into molecular markers in the context of screening programs. More and more miRNA signatures associated with cervical cancer will be discovered as result the commercial availability of several miRNA array platforms with varying quality and sensitivities [111,112]. Furthermore, HPV infection, viral genotype, physical viral DNA state, and carcinogenic risk all affect changes to the methylation status of cellular DNA. Since miRNAs linked to treatment resistance in cervical cancer, new combination therapy involving miRNA inhibitors, or there addition to chemotherapy or radiotherapy may be created. These findings point to varieties of molecular targeted therapy and miRNA-specific customized therapies, and miRNAs anticipated to play a significant role in detection and management in cervical cancer.

## Data availability statement

Data included in article/supplementary material/referenced in article.

## CRediT authorship contribution statement

**Hiwot Tezera Endale:** Writing – review & editing, Writing – original draft, Visualization, Validation, Supervision, Software, Resources, Project administration, Methodology, Investigation, Funding acquisition, Formal analysis, Data curation, Conceptualization. **Yitbarek Fantahun Mariye:** Formal analysis, Conceptualization. **Habtu Kifle Negash:** Investigation, Data curation, Conceptualization. **Fethiya Seid Hassen:** Data curation, Conceptualization. **Wastina Bitewlign Asrat:** Data curation, Conceptualization. **Tiget Ayelgn Mengstie:** Data curation, Conceptualization. **Winta Tesfaye:** Writing – review & editing, Supervision, Methodology, Investigation, Formal analysis, Data curation, Conceptualization.

## Declaration of competing interest

The authors declare that they have no known competing financial interests or personal relationships that could have appeared to influence the work reported in this paper.
